# Covalent Anchoring of Chloroperoxidase and Glucose Oxidase on the Mesoporous Molecular Sieve SBA-15

**DOI:** 10.3390/ijms11020762

**Published:** 2010-02-24

**Authors:** Dirk Jung, Carsten Streb, Martin Hartmann

**Affiliations:** Erlangen Catalysis Resource Center, Universität Erlangen Nürnberg, Egerlandstr. 3, 91058 Erlangen, Germany; E-Mails: dirk.jung@ecrc.uni-erlangen.de (D.J.); carsten.streb@chemie.uni-erlangen.de (C.S.)

**Keywords:** functionalization, mesoporous silica, enzyme immobilization

## Abstract

Functionalization of porous solids plays an important role in many areas, including heterogeneous catalysis and enzyme immobilization. In this study, large-pore ordered mesoporous SBA-15 molecular sieves were synthesized with tetraethyl orthosilicate (TEOS) in the presence of the non-ionic triblock co-polymer Pluronic P123 under acidic conditions. These materials were grafted with 3-aminopropyltrimethoxysilane (ATS), 3-glycidoxypropyltrimethoxysilane (GTS) and with 3-aminopropyltrimethoxysilane and glutaraldehyde (GA-ATS) in order to provide covalent anchoring points for enzymes. The samples were characterized by nitrogen adsorption, powder X-ray diffraction, solid-state NMR spectroscopy, elemental analysis, diffuse reflectance fourier transform infrared spectroscopy and diffuse reflectance UV/Vis spectroscopy. The obtained grafted materials were then used for the immobilization of chloroperoxidase (CPO) and glucose oxidase (GOx) and the resulting biocatalysts were tested in the oxidation of indole. It is found that enzymes anchored to the mesoporous host by the organic moieties can be stored for weeks without losing their activity. Furthermore, the covalently linked enzymes are shown to be less prone to leaching than the physically adsorbed enzymes, as tested in a fixed-bed reactor under continuous operation conditions.

## 1. Introduction

Aiming at the preparation of stable biocatalysts, permanent immobilization and encapsulation of enzymes on solid inorganic materials have been the focus of intense studies due to potential applications in biocatalysis [1]. The controlled fabrication of nanometer-scale objects for selected applications is another area of increasing interest [2]. For industrial use, an efficient recovery and reuse of costly enzymes is essential. Moreover, immobilization enables the use of enzymes in flow-type fixed-bed reactors. In addition the storage and operational stability is enhanced, *viz*. unfolding, denaturation by heat or organic solvents or by autolysis is suppressed [3].

There have been many studies devoted to enhancing the catalytic performance of enzymes by immobilization. For example the immobilization of penicillin G acylase (PGA; EC 3.5.1.11) [4–7], which catalyzes the deacylation of penicillin G to 6-aminopenicillanic acid, an important intermediate in the manufacture of semisynthetic penicillin and lipase (triacylglycerol ester hydrolase, EC 3.1.1.3) [8–11], which has been widely used in various biotechnological applications is subject of great interest. To date a number of different enzymes has been immobilized onto functional mesoporous supports [4–22]. Recently, Bein and co-workers presented the covalent immobilization of trypsin onto SBA-15. An azide-functionalized mesoporous silica (SBA-N_3_) was used as support which was reacted with an acetylene-modified trypsin in a copper(I)-catalyzed Huisgen reaction (“click” reaction) [22]. One of the most versatile enzymes, chloroperoxidase (CPO) from *Caldariomyces fumago*, has been the subject of intensive research over the last years. Two main goals have to be achieved for preparing immobilized CPO for industrial applications: a) to circumvent deactivation and b) to avoid leaching. In our previous publication, we have shown that the deactivation of CPO can be suppressed by generating the required oxidant hydrogen peroxide *in-situ* from glucose in a tandem-reaction with immobilized glucose oxidase (GOx) [23,24]. Leaching can be prevented by a strong and permanent binding between the enzyme and the carrier. In general, physical bonding is too weak to keep the enzyme remnant to the carrier under industrial conditions of high reactant and product concentrations. On the other hand, covalent binding of the enzyme to the support has the advantage that interaction of the enzyme with the support is increased and leaching is reduced. Aburto *et al*. immobilized CPO covalently on functionalized SBA-16 and reported an increase in stability compared with free CPO in the presence of the denaturant urea [25]. Montiel *et al*. tested covalently bonded chloroperoxidase on SBA-15 in the oxidation of 4,6-dimethyldibenzothiophene (4,6-DMDBT) [26]. Hudson *et al*. developed mesoporous materials functionalized with amine groups as potential supports for CPO [27]. A periodic mesoporous organosilane with pore entrances large enough to allow the enzyme enter the pores was found to be a good support and can be reused 20 times with retention of activity. Mesoporous structures can be functionalized with organic moieties, a topic which was investigated intensively over the last years. An easy access to organic-inorganic hybrid materials based on SBA-15 is provided by surface modification [28,29]. Mesoporous materials can be functionalized by postmodification (grafting) and direct synthesis (co-condensation) [30,31]. Grafting is the more common method where surface modification is achieved by covalent linking of organosilane species with surface silanol groups. However, the grafting method has several shortcomings, *i.e.*, the pore size is reduced due to the attachment of a layer of functional moieties on the surface and the number of accessible surface silanol groups on the mesoporous silica materials is reduced. Co-condensation allows surface modification of the mesoporous materials in a single step by copolymerization of organosilanes with silica or organosilica precursors in the presence of a surfactant [32,33]. The disadvantage of this method is that the functional groups may not be homogenously distributed. In contrats, grafting is easily feasible for a broad range of functional groups and does not interfere with the formation of the silica framework. Especially, in the field of enzyme immobilization, grafting discloses directly the influence of the incorporated functional groups because supports featuring the same textural properties can be functionalized with different organomoieties. A variety of functional groups has been incorporated into mesoporous materials such as aliphatic hydrocarbons, thiol groups, vinyl groups, phenyl groups, amine groups and perfluoro groups [20,31]. SBA-15 has been functionalized e.g., with amine, thiol, nitrile, phenyl, and chloro groups [20]. Chong *et al*. found that surface modification using 3-aminopropyl triethoxysilane, producing a terminal amine group (-NH_2_) is useful for covalent coupling of proteins to the surface of silica materials [34]. Nevertheless, several drawbacks have to be considered. Since this method implements a chemical reaction, a certain activation energy is needed to promote the bond formation. Hence, often high temperatures have to be applied, which might result in deactivation of the enzyme [5,35]. On the other hand, the application of a comparatively reactive linker might lead to multiple connections to a particular enzyme [28,35]. Thereby, the quaternary structure of the enzyme is heavily distorted resulting in a dramatic decrease in enzymatic activity.

**Scheme 1.**
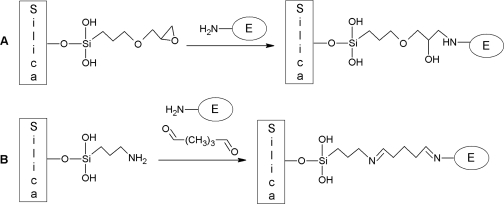
Covalent enzyme immobilization: (A) epoxy-modified silica reacts with the *N*-terminus of the enzyme; (B) aminopropyl-modified silica reacts with glutaraldehyde and the N-terminus of the enzyme.

In this work, we examine the grafting method for the functionalization of mesoporous SBA-15 materials with 3-glycidoxypropyltrimethoxysilane (Scheme 1A) and 3-aminopropyl trimethoxysilane (Scheme 1B) [36]. In a subsequent step, the aminopropyl moiety is reacted with glutaraldehyde. We want to anchor chloroperoxidase (CPO) and glucose oxidase (GOx) to the carrier surface via chemical covalent bonding for to immobilize the enzyme permanently without the danger of leaching (see Scheme 1).

Scheme 1(A) illustrates the enzyme immobilization on amino-modified silica [35–40]. A bifunctional spacer, glutardealdehyde, connects the amino function of the enzyme with the pore wall *via* two imino moieties. In Scheme 1(B) the enzyme immobilization on epoxy-modified silica is shown. The epoxy function of the organo-modified silica alkylates the amino function of the enzyme. Due to its high reactivity, other nucleophils like hydroxyl or thiol moieties can also react with the epoxy group, hence, the selectivity to exclusively link specific moieties is low. Furthermore, we have shown that covalently bonded CPO and GOx on the mesoporous material SBA-15 exhibit a higher operational stability in a continuously operated fixed-bed reactor compared to a catalyst prepared by physisorption of the respective enzymes.

## 2. Results and Discussion

**Figure 1.**
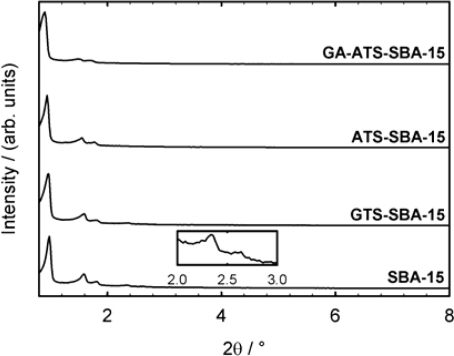
Powder X-ray diffraction patterns of calcined SBA-15 and the modified SBA-15 materials.

Figure 1 depicts the XRD powder patterns of siliceous SBA-15 after different modification steps in comparison to the parent material. Typically, four to five separate Bragg reflections are detected depending on the long range order of the material. The signals can be indexed according to the Miller indices (hkl): An intensive signal is detected for the (100) reflection. At slightly higher 2θ values, low intensity Bragg reflections of (200) and (110) planes as well as (210) and (300) indicative of a high-quality well-ordered material were also detected. In agreement with previous studies, the diffractograms indicate a well-ordered 2-dimensional array of hexagonally arranged pores [43,44].

The diffractograms confirm that the hexagonal structure was not affected by the post-synthesis grafting. Only minor alterations of the unit cell size a_0_ due to the modification were observed (Table 1).

**Figure 2.**
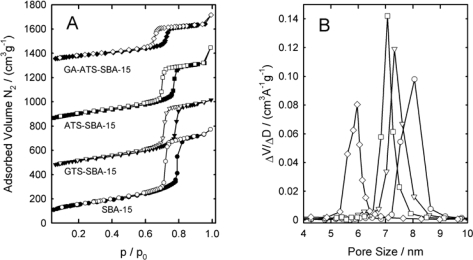
Nitrogen sorption isotherms at 77 K of SBA-15(circle), GTS-SBA-15(triangle), ATS-SBA-15(square) and GA-ATS-SBA-15(rhombus). Isotherms are shifted by 500 cm^3^g^−1^ for clarity. Closed symbols: adsorption branch, open symbols: desorption branch. B: BJH Pore size distribution of the samples calculated from the desorption branch of the isotherm.

Figure 2(A) shows the nitrogen adsorption isotherms at 77 K of SBA-15, GTS-SBA-15, ATS-SBA-15 and GA-ATS-SBA-15. All isotherms are of type IVa according to the IUPAC classification and show a H1 hysteresis loop [45,46]. Figure 2(B) depicts the pore size distribution of the four materials determined from the desorption branch of the particular isotherm using the BJH formalism [42]. As expected, specific pore volume and pore diameter (as indicated by the decreasing height of the capillary condensation step) decrease with the introduction of larger organic moieties. In Table 1, the textural properties of the parent SBA-15 are compared with those of the modified SBA-15 materials. As a consequence of the introduction of the space-filling ATS ligands, the pore diameter decreases from 8.0 nm for SBA-15 to 7.0 nm for ATS-SBA-15. Further reaction of the amino function with glutaraldehyde results in a further decrease of the pore diameter to 6.0 nm (GA-ATS-SBA-15). The hybrid material GTS-SBA-15 exhibits a pore diameter of 7.3 nm due to the grafted organic epoxy moieties. However, the pore diameter of the GTS-modified material is larger compared to ATS-SBA-15 suggesting a lower number of grafted organic moieties.

To corroborate these results, the surface coverage α of the organic ligands based on the carbon content was calculated as described by Jaroniec *et al*. [47]:

(1) $$\alpha =\frac{10^{6}}{A_{\text{BET}}\left[M_{\text{Carbon}}100(\frac{n_{C}}{P_{C}})-M_{\text{Ligand}}\right]};\;[\alpha ]=\mu \text{mol}\;{\text{m}}^{-2}$$

The surface coverage α of the organic ligands is calculated from the carbon content P_C_, the molar mass of carbon M_Carbon_, the number of carbon atoms in the ligand n_C_ and on the molar mass of the ligand M_Ligand_ from the elemental analysis.

Table 2 shows the calculated composition of the organic moieties and the elemental analysis of GA-ATS-SBA-15. For ATS-SBA-15, a carbon content of 9.3 wt.-% was found by chemical analysis. Using equation (1) a surface coverage of 7.7 μmol m^−2^ is calculated. A carbon content of 3.93 wt.-% was determined for GTS-SBA-15, which translates into a surface coverage of 1.3 μmol m^−2^. The lower surface coverage determined for the GTS modified SBA-15 supports the results of the N_2_ sorption experiments and indicates that only a partial conversion of the surface silanol groups was achieved. A carbon content of 19.6 wt.-% was determined for GA-ATS-SBA-15, which corresponds to a surface coverage of 9.7 μmol m^−2^. The calculated chemical composition of GA-ATS is somewhat different to the experimental value determined for GA-ATS-SBA-15, which implies that not all amino groups are oxidized by glutaraldehyde to an imino group. Assuming a GA-ATS/ATS ratio of 2/1 the calculated elemental contents are in excellent agreement with the experimental values. Thus, only two out of three amino functions react with glutaraldehyde to give an imino function.

**Figure 3.**
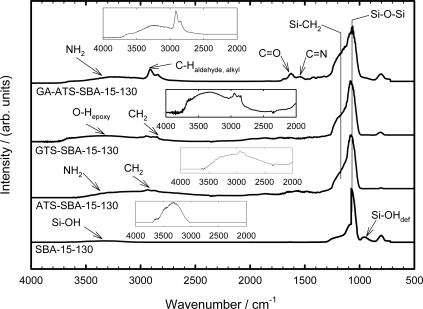
DRIFT spectra of SBA-15, ATS-SBA-15, GTS-SBA-15 and GA-ATS-SBA-15. The suffix -130 refers to the synthesis temperature of the parent material.

Diffuse reflectance Fourier transform infrared spectroscopy (DRIFT) is used to identify functional groups of the modified silica material. The DRIFT spectra of SBA-15 before and after modification with 3-aminopropyltrimethoxysilane (ATS), 3-glycidoxypropyltrimethoxysilane (GTS) and glutaraldehyde (GA) are shown in Figure 3. The modified samples exhibit a strong band at a wave number of 1085 cm^−1^ due to Si–O–Si vibrations and of a shoulder at about 1200 cm^−1^, which is ascribed to Si–CH_2_ vibrations. The broad signal between about 3600 cm^−1^ and 3000 cm^−1^ is due to adsorbed or hydrogen-bonded water molecules. At 962 cm^−1^, a characteristic silanol deformation mode is found, which is only observed for the parent SBA-15 material and disappears after surface modification. The amino-functionalized material ATS-SBA-15 exhibits a band at 2925 cm^−1^ which is ascribed to the C–H stretching mode of the CH_2_ groups in the propyl chain [48–50]. The C–N stretching of the aminopropyl moiety vibration is typically observed at wavenumbers between 1000 and 1200 cm^−1^ [50]. However, this band is not resolved due to the overlap with the Si–O–Si IR absorptions in the range of 1130 to 1000 cm^−1^ and of the Si–CH_2_ vibrations between 1250 and 1200 cm^−1^. At about 3300 cm^−1^, the N–H stretching mode of the amino groups is observed.

After modification of SBA-15 with 3-glycidoxypropyltrimethoxysilane, a broad band between 3580 and 3000 cm^−1^ associated with O-H as well as epoxy stretching vibrations is observed. The O-H signal is associated with hydrolyzed epoxy functions as well as Si-OH functions, indicating that not all of the surface silanol moieties were converted. At about 2925 cm^−1^, a C–H stretching mode is detected as well. The glutaraldehyde-modified ATS-SBA-15 exhibits strong bands at 2950 and 2880 cm^−1^ [50]. These bands are ascribed to aldehyde C–H stretching vibrations and alkyl C–H stretching vibrations, respectively. C=O and C=N stretching modes are detected at 1666 cm^−1^ and 1580 cm^−1^, respectively. Noticeably, the band at 3300 cm^−1^ representing the N–H stretching mode of the amino groups is detected as well. This implies that not all amino moieties of the ATS-functionalized silica have reacted with glutaraldehyde, which is in line with N_2_ adsorption and chemical analysis (see above). The silanol deformation band at 960 cm^−1^ is not observed in the spectra of the modified SBA-15 materials giving further evidence for the effective surface modification.

**Figure 4.**
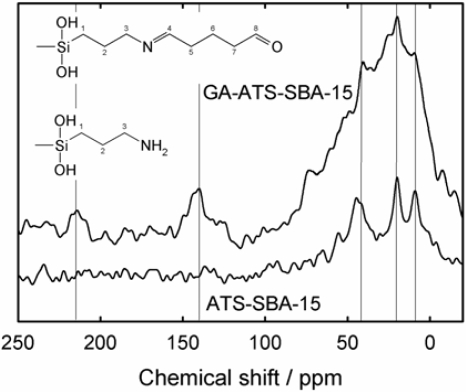
^13^C-CP-MAS-NMR spectra of ATS-SBA-15 and GA-ATS-SBA-15.

Figure 4 shows the solid-state ^13^C-MAS-NMR spectra of the grafted ATS-SBA-15 and GA-ATS-SBA-15 materials. The ATS functionalized SBA-15 exhibits resonances with chemical shifts of δ = 8, 19 and 40 ppm. These resonances are assigned to different carbons in the organosilane moieties denoted as C^1^, C^2^ and C^3^ (see Figure 4) [30]. The observation of these resonances for the material GA-ATS-SBA-15 can be related to the same carbons. The not well resolved signal between δ = 0 and 75 ppm also includes resonances of the carbons C^5^, C^6^ and C^7^. The signals at δ = 140 and 215 ppm are assigned to C^4^ (C=N) and C^8^ (C=O), respectively. The weak signal and the low signal/noise ratio are indicative for the incomplete functionalization of the silanol groups by ATS. Moreover, only two-thirds of the ATS groups react to GA-ATS moieties, which is in line with the elemental analysis. In addition, ^29^Si MAS NMR data also support the successful modification of SBA-15 (not shown).

**Figure 5.**
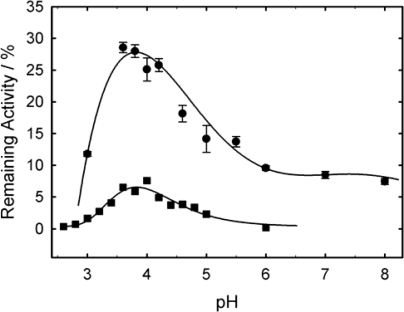
Influence of the pH value of the adsorption solution on the activity of CPO (▪) and GOx (•) adsorbed on SBA-15. Activity values are given in percent of initial CPO activity in solution which was determined using the MCD assay as described in 3.2.

Adsorption studies were performed in order to determine the optimum pH value for batch adsorption of GOx and CPO on unmodified SBA-15. Figure 5 shows the activity of GOx and CPO adsorbed on SBA-15 as a function of the pH value of the solution from which the enzyme was adsorbed onto the support. The maximum activity, viz. the highest enzyme loading, is found for solutions possessing pH values of 3.5 and 4.5. The retained activity amounts to about 7% for CPO and 30% for GOx, respectively. The optimum pH value for enzyme adsorption is 3.8 for CPO and 4.0 for GOx. These pH values are close to the isoelectric points (pI) of the enzymes, which are 4.0 (CPO) and 4.2 (GOx), respectively [38]. In contrast, the pH level of maximum CPO activity ranges from 5 to 6, while the maximum GOx activity is detected between pH 6.8 and 7.8 employing standard activity assays. However, in order to increase the enzyme loading and to avoid leaching of the enzymes under reaction conditions, it is desirable to anchor the enzymes covalently to the mesoporous support. For the experiments with the modified supports, the solution pH values were varied only between 3.6 and 4.2.

**Figure 6.**
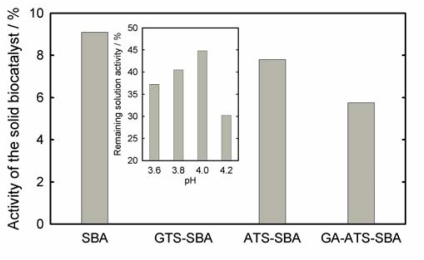
Activity of the covalent immobilized chloroperoxidase on SBA-15 and modified materials expressed in percent of the initial solution activity (pH = 3.8). Insert: covalent immobilization of chloroperoxidase over GTS-SBA-15. Activity values are given in percent of initial CPO activity as determined by the MCD assay (see Section 3.2).

**Figure 7.**
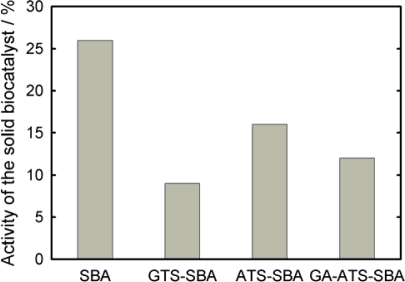
Activity of the covalent immobilized glucose oxidase on SBA-15 and modified materials expressed in percent of the initial solution activity (pH = 4.0), determined as described in the Experimental section.

First of all, the SBA-15 material functionalized with epoxy moieties (GTS-SBA-15) was tested for immobilization of CPO. After the adsorption experiment, the remaining activity of the CPO solution varies between 30 and 45% depending on the pH value of the solution (insert of Figure 6). Therefore, assuming that no deactivation of the CPO takes place, it is found that a significant amount of CPO is immobilized on the support. However, the activity observed for the immobilized CPO is close to zero suggesting that the epoxy moiety is too reactive and probably binds to several sites of the enzyme. We assume that the sensitive quaternary structure is disturbed to a significant extent. Consequently, the chloroperoxidase, which is covalently linked to epoxy moieties, is deactivated. In conclusion, the studies with GTS-SBA-15 showed that a too reactive linker results in large amounts of immobilized but inactive CPO. On the other hand, chloroperoxidase immobilization into SBA-15 shows a remaining activity of about 9%, ATS-SBA-15 of 7% and GA-ATS-SBA-15 of 6%, respectively (Figure 6). The decrease in activity is mainly due to the fact that fewer CPO molecules can be immobilized because of the reduced pore sizes which come along with the functionalization. In contrast, Figure 7 shows the activity of GOx anchored to SBA-15 functionalized by different routes in comparison to GOx adsorbed onto SBA-15. When the anchoring is realized via the epoxy function, the resulting heterogeneous catalyst possesses an activity of about 9% of the initial GOx activity in solution.

In comparison to the immobilization of CPO, which results in a complete loss of activity, the results of GOx immobilization on GTS-SBA-15 imply that successful covalent anchoring depends not only on the chemical linker employed but also on sensitivity of the quaternary structure of the enzyme used. The observed decrease of activity of GOx-GA-ATS-SBA-15 compared to GOx-ATS-SBA-15 might be due to the covalent anchoring but might also be a consequence of the decreasing pore diameter and pore volume of the support as a consequence of surface functionalization. Thus, the implementation of the bifunctional linker glutaraldehyde in order to create a bond between the amino-functionalized ATS-SBA-15 and the enzyme N-terminus, e.g., with the three lysine groups on the enzyme surface (Lys 112, Lys 145 and Lys 211), was found to be a more promising approach. We also observed that the storage of CPO immobilized into GA-ATS-SBA-15 in a buffer at pH = 3.4 results in leaching of the enzyme from the support due to hydrolytic cleavage of the imino bond. Storage at pH = 7.0 resulted in complete loss of enzyme activity after a few days. Thus, the pH was adjusted to 5.0 in order to minimize both, the enzyme deactivation as well as bond cleavage. Immobilization of glucose oxidase into GA-ATS-SBA-15 at pH = 4.0 and subsequent washing with a buffer solution (pH = 3.0) results in a heterogeneous catalyst with a remaining activity of about 12%. Drying of the washed material in air until a paste-like solid is obtained, results in a catalyst, which is stable over weeks without a significant loss in activity.

**Figure 8.**
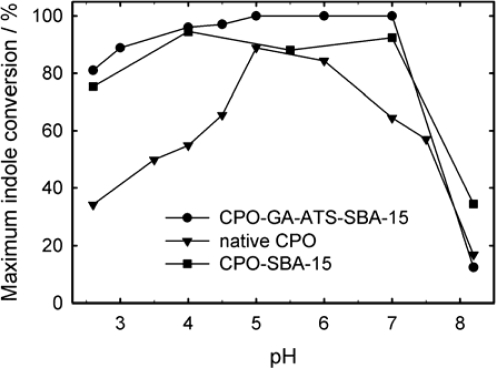
Indole oxidation over CPO-GA-ATS-SBA-15 (circle), CPO-SBA-15 (square) and native CPO (triangle) as a function of the pH of the reaction mixture. Catalyst amount: 2.5 U; T_R_ = 20 °C; substrate: indole (5 mL, 3.5 mM); oxidant: H_2_O_2_ (1.95 mL, 8.8 mM, flow rate = 15 μL min^−1^).

The catalytic performances of covalently immobilized CPO-GA-ATS-SBA-15 and adsorbed CPO-SBA-15 were compared at various pH values between 2.6 and 8.2 in order to obtain information on the overall activity and influence of the pH of the reaction mixture on the activity of the supported enzyme catalyst (Figure 8). The experiments with covalently immobilized CPO showed significantly higher conversions compared to physically adsorbed CPO-SBA-15. At pH values between 3 and 7, the conversion is larger than 80% for CPO-GA-ATS-SBA-15 and is close to 100% at pH values from 5 to 7. Over the whole pH range studied, the maximum conversion over CPO-SBA-15 and native CPO is always lower compared to the catalyst compared by covalent anchoring. Morris and Hager reported that the optimum pH value for native CPO ranges from 5 to 6 [40]. Our experiments suggest that on SBA-15 immobilized CPO exhibits an expanded range of optimum performance at pH values between 4 and 7.

**Figure 9.**
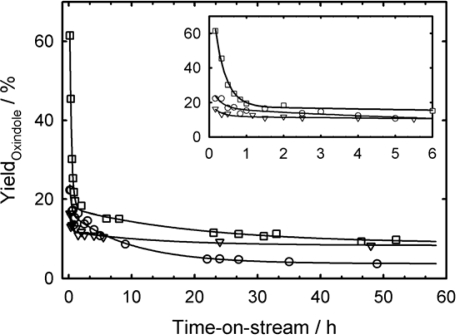
Tandem reaction with 150 U CPO-GA-ATS-SBA-15 and 7 U GOx-GA-ATS-SBA-15 (triangle), 150 U CPO-SBA-15 and 7 U GOx-SBA-15 (circle), 265 U CPO-GA-ATS-SBA-15 and 10.5 U GOx-GA-ATS-SBA-15 (square) as catalysts in the indole oxidation. T_R_ = 20 °C; substrate: indole (3.5 mM), glucose (50 mM), flow rate = 0.5 mL min^−1^, average particle size = 2 μm in (as determined by ESEM).

In our previous study, we reported that the deactivation of CPO in the catalytic oxidation of indole is reduced when the required hydrogen peroxide is generated *in-situ* by d-glucose oxidation using immobilized GOx in a batch reactor [24]. Here, we have studied the stability of the solid catalysts of the tandem system in a flow-type fixed-bed reactor, which allows to study catalyst deactivation and/or leaching as a function of time-on-stream. Figure 9 compares the fixed-bed reactor performance of the covalently immobilized with the adsorbed enzymes. For CPO and GOx adsorbed onto SBA-15 a decrease of the oxindole yield with time-on-stream is detected. For the catalyst bed, consisting of 150 U CPO-SBA-15 and 7 U GOx-SBA-15, the initial oxindole yield amounts to 16%. After 48 h time-on-stream, the 2-oxindole yield amounts to 3.5%. In contrast, the immobilized enzymes catalyst shows a completely different performance. In the first two hours, the oxindole yield is reduced from 61% over 265 U CPO-GA-ATS-SBA-15 and 10.5 U GOx-GA-ATS-SBA-15 to about 10% after more than 48 hours. Then a roughly constant yield is reached much earlier as in case of the adsorbed enzymes. Thus, we have to conclude that even functionalized SBA-15 materials contain a certain amount of only physisorbed enzymes. This weak interaction between the mesoporous host and the enzyme results in rapid leaching of the enzyme during the first few hours of time-on-stream, which is indicated by a strong decrease of the oxindole yield. However, by using immobilized CPO and GOx as catalysts in the tandem system of indole oxidation, the final oxindole yield amounts to 8.3% (9.4% for 265 U CPO-GA-ATS-SBA-15 and 10.5 U GOx-GA-ATS-SBA-15), which is more than twice the amount compared to the physisorbed enzymes at similar catalyst loading.

## 3. Experimental Section

### 3.1. Synthesis of SBA-15 Grafted with ATS, GTS and GA

SBA-15 was synthesized at 130 °C according to the procedure outlined in our previous publication [39]. Prior to the grafting step, 1.0 g of SBA-15 was activated at 150 °C for 24 h under vacuum. The material was then transferred to a round bottom flask equipped with a stopcock and condenser. Fifty mL of dry toluene and 2.0 mL (1.892 g, 8.55 mmol) of 3-aminopropyltrimethoxysilane (ATS) or 2.5 mL (2.675 g, 11.32 mmol) of 3-glycidoxypropyltrimethoxysilane (GTS) were added under nitrogen. The reaction mixture was refluxed under magnetic stirring for 24 h. After cooling, the solid product was recovered by suction filtration. Washing with toluene (100 mL) and acetone (100 mL) yielded a white solid that was dried under vacuum for 3 h and stored under argon. The recovered materials are referred to as ATS-SBA-15 and GTS-SBA-15, respectively.

ATS-SBA-15 (500 mg) was mixed with 30 mL of an aqueous 1 wt.-% glutaraldehyde (GA) solution and stirred for 4 h. Immediately after mixing, the suspension turned yellow, later orange and red. After 4 h, the product was recovered by suction filtration over a filter funnel equipped with a D3 frit. Washing with 500 mL water yielded a red solid that was dried under vacuum. The recovered material is referred to as GA-ATS-SBA-15.

### 3.2. Immobilization of CPO and GOx

Ten mg of the functionalized SBA-15 materials and 10 or 5 units (U) of CPO (GOx) were suspended in 5 mL of a 50 mM aqueous citrate buffer at various pH values. Details of the procedure for the unmodified SBA-15 support are reported in our previous publication [39]. The obtained solid materials were referred to as CPO (GOx)-X-SBA-15 (X = ATS, GTS, GA-ATS). After assaying the solid material for activity, the suspension was stored at 4 °C. Leaching was tested by assaying the supernatant solution of the suspension for activity after storing for several days. For the CPO-X-SBA-15 materials, the standard MCD activity assay, viz. the chlorination of monochlorodimedone (MCD) to dichlorodimedone (DCD) [40,41], was performed. The activity of the GOx-X-SBA-15 materials was tested as follows: To 5 mL of an acidified *β*-d-glucose solution (0.1 mM), potassium iodide was added in excess. Thereafter, an aliquot of the GOx solution or suspension was added. During the reaction the iodide is oxidized by the formed H_2_O_2_ to iodine. Thereby, the color of the reaction mixture turns red. The iodine concentration was determined photometrically at a detection wavelength of 352 nm. This activity test was performed in a buffer solution (pH = 5.5) at 25 °C. The amount of iodine formed is equivalent to the amount of H_2_O_2_ produced by GOx. 1 unit (U) of GOx is defined as the amount of GOx that oxidizes 1 μmol *β*-d-glucose to 1 μmol H_2_O_2_ per minute (1 U GOx ≡ 1 μmol H_2_O_2_ min^−1^). Enzyme activities given in U generally refer to the activity measured by the assays described above.

### 3.3. Characterization of the Mesoporous Materials

All materials were characterized by X-ray powder diffraction (Siemens D5005) using monochromatic CuKα-radiation with a wavelength of λ_CuKα_ = 0.15405 nm. The nitrogen adsorption experiments were performed in a Quantachrome Autosorb 1 instrument at liquid nitrogen temperature (77 K). Samples were degassed for 12 h at 10^−5^ hPa at a temperature of 250 °C. The specific surface area was determined in the range of p/p_0_ values between 0.05 and 0.2 using the multiple-point *Brunauer-Emmett-Teller* (BET) method. The specific pore volume V_Pore_ was obtained from the adsorption isotherm after capillary condensation was completed (p/p_0_ ≈ 0.90). The pore size distribution was determined using the BJH method (developed by Barrett, Joyner and Halenda) [42]. For the BJH calculations, the desorption branch of the nitrogen isotherm was used. The infrared spectra of the mesoporous materials were measured by the diffuse reflectance technique using a Nicolet Nexus FT-IR spectrometer equipped with a SpectraTech Diffuse Reflectance Accessory with dried KBr as background. CHN elemental analysis was carried out employing a Perkin Elmer 2400 CHN Elemental Analyzer. Solid-state ^13^C magic-angle spinning (MAS) nuclear magnetic resonance (NMR) spectra were recorded at a resonance frequency of 100.6 MHz on a Varian AS400 NMR using cross polarization and high power decoupling; 5 mm rotors were spun at 6 kHz. Adamantane was used as a reference.

### 3.4. Indole Oxidation

**Scheme 2.**
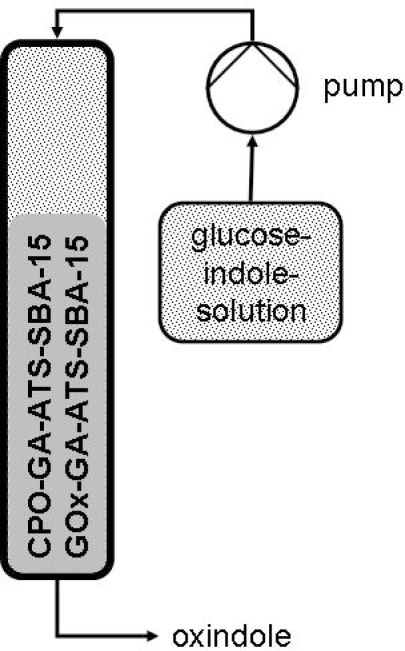
Schematic presentation of the fixed-bed reactor.

In a batch reactor, 2.5 U of the heterogeneous CPO catalyst were suspended in 5 mL of a saturated aqueous buffered indole solution (3.5 mM, 18 μmol indole) and allowed to equilibrate for 5 min [38]. A hydrogen peroxide (8.8 mM) solution was then added dropwise for 150 min at a flow rate of 15 μL·min^−1^. For the continuous flow experiments, a fixed-bed reactor was filled with 150 U CPO catalyst and 7 U GOx catalyst. A buffered indole (3.5 mM) glucose (50 mM) solution (pH = 5.5) was pumped through the reactor at a flow rate of 0.5 mL·min^−1^ and a pressure of about 250 bar (Scheme 2).

## 4. Conclusions

It is shown that the described grafting methods allow the functionalization of the mesoporous silica SBA-15 with different organic moieties. 3-glycidoxypropyltrimethoxysilane (GTS) and 3-aminopropyltrimethoxysilane (ATS) are used as grafting compounds, further treatment of the 3-aminopropyl modified material with glutaraldehyde (GA) results in GA-ATS-SBA-15. The modified mesoporous silica supports are characterized by DRIFT spectroscopy and elemental analysis. Furthermore, the elemental analysis suggests that two of three amino moieties react with glutaraldehyde to the imino moiety of GA-ATS-SBA-15. Obviously, covalent immobilization has to be performed in such a manner that the quaternary structure of the immobilized enzyme is not heavily distorted. The GTS-SBA-15 material adsorbed chloroperoxidase as well as glucose oxidase but only GOx supported on the 3-glycidoxypropyl modified SBA-15 is further active. GA-ATS-SBA-15 was successfully used as a covalent anchoring host for chloroperoxidase and glucose oxidase. The catalyst is stable for weeks without losing any activity, while physisorption on SBA-15 shows a decrease of activity after a certain period of time. The heterogenized biocatalysts CPO-GA-ATS-SBA-15 and CPO-SBA-15 are better oxidation catalysts compared to native CPO. Both show an increased optimum activity range between pH = 4 and 7 as well as higher maximum conversions throughout the whole pH range studied. Thereby, a lower susceptibility to oxidative deactivation for immobilized CPO was found which results in an increased stability. Furthermore, we have shown that leaching can be circumvented by linking the enzymes covalently to the support even under harsh conditions (250 bars in a plug-flow reactor). The final yield in indole catalysis is about two times higher for CPO-GA-ATS-SBA-15 as compared to CPO-SBA-15 due to the higher stability of the former catalyst. By performing this tandem reaction with covalently linked CPO and GOx two main technical difficulties in heterogeneous biocatalysis are addressed: i) the deactivation of the immobilized CPO is circumvented by *in-situ* hydrogen peroxide generation [24,39] and ii) enzyme leaching is suppressed by functionalization of the porous host followed by covalent anchoring of the enzymes to the support.

## Figures and Tables

**Table 1.** Textural properties of the synthesized mesoporous supports: pore diameter (d_P_), BET surface area (A_BET_), pore volume (V_P_) and unit cell parameter (a_0_).

**Sample**	**d_P_/ nm**	**A_BET_/ (m^2^g^−1^)**	**V_P_/ (cm^3^g^−1^)**	**a_0_/ nm**
SBA-15	8.0	530	1.05	12.1
GTS-SBA-15	7.3	433	0.90	11.1
ATS-SBA-15	7.0	392	0.79	11.3
GA-ATS-SBA-15	6.0	294	0.53	11.3

**Table 2.** Calculated elemental composition of the different moieties in comparison to the elemental analysis of GA-ATS-SBA-15.

**Moities (calculated)**	**Elemental content/wt.-%**			**Molecules/nm^2^**
	**C**	**H**	**N**	
ATS	9.3	2.1	2.2	4.7
GA-ATS	19.6	3.4	2.1	
GA-ATS/ATS = 2/1	16.9	3.0	2.1	
Sample (observed)				

GA-ATS-SBA-15	16.57	2.79	2.09	5.9
